# The Role of Sexually Transmitted Infections in Police as Clients Among Street-Based Female Sex Workers in Baltimore City

**DOI:** 10.1097/OLQ.0000000000001292

**Published:** 2020-09-15

**Authors:** Susan G. Sherman, Danielle Friedman Nestadt, Bradley E. Silberzahn, Michele Decker, Ju Nyeong Park, Katherine H.A. Footer

**Affiliations:** From the Departments of ∗ Health, Behavior and Society; † Population, Family and Reproductive Health, Johns Hopkins Bloomberg School of Public Health, Baltimore, MD

## Abstract

Research among street-based female sex workers (FSWs) has documented many harms caused by police. One harm that has received little attention is that of police as clients. We examined this interaction in a 12-month longitudinal cohort study of street-based FSWs in Baltimore, MD.

**Methods:**

We explored longitudinal bivariate and multivariate associations between having police clients and independent variables that focused on sexually transmitted infections (STIs), as well as demographic, structural, substance use, police interaction, and violence-related factors.

**Results:**

Mean participant age was 35.8 years, 65.9% were White, and more than half (53.3%) had less than a high school education. Most (70.3%) used heroin daily, and 24.8% reported having police as clients over the study period. In a multivariate model, factors independently associated with recent police clients were recent arrest (adjusted odds ratio [aOR], 1.76; 95% confidence interval [CI], 1.03–2.99; *P* = 0.037), coerced or forced sex by police (aOR, 4.47; 95% CI, 1.79–11.12; *P* = 0.001), higher number of egregious police practices experienced (aOR, 1.77; 95% CI, 1.38–2.29; *P* < 0.001), and prevalent STI infection (aOR, 2.43; 95% CI, 1.46–4.04; *P* = 0.001).

**Conclusions:**

The study uniquely documents both the frequency with which street-based FSWs take police as clients and the role of egregious police practices and prevalent STIs in association with police as clients. Results indicate the police-as-client association as a form of “everyday violence,” which both normalizes and legitimizes police power and structural violence. Alongside the urgent need for decriminalization of sex work and STI prevention programs tailored for this complex population, prompt investigation and harsher penalties for police officers who engage in sex with FSW could help shift police culture away from abuse.

## Abstract 2

A study of street-based female sex workers in Baltimore, Maryland, found that prevalent sexually transmitted infections, recent arrest, and coerced/forced sex by police were associated with having police officers as sex work clients.

Globally, female sex workers (FSWs) experience a high burden of sexually transmitted infections (STIs) such as chlamydia, gonorrhea, and trichomonas, although studies in the United States are rare.^1–3^ In the context of criminalization, FSWs' risk is characterized by broad social and structural environmental factors extending beyond individuals' behavior or volition.^4,5^ In particular, elevated rates of violence, STIs, and HIV have been linked to both lawful (e.g., arrest and move-along tactics) and unlawful (e.g., sexual and physical abuse) policing practices.^1,6–8^ The literature in this domain has advanced the operationalization of key police determinants implicated in FSWs' STI and HIV risk.^4,9–12^

An aspect of FSW-police interaction that has received little attention in the literature is the police-as-client interaction, whereby police pay FSW for sex. Police-as-client has been described as distinct from coerced sex and characterized more as a “choice,” suggesting FSWs potentially have more power over their interactions and the possibility of reduced situational vulnerability in a context of constrained choices.^11,13^ However, threat of arrest is always present, even when not expressed explicitly. Therefore, declining sex can be seen as riskier, with decreased autonomy and agency in negotiations regarding payments, sex acts, and condom use.^11,13,14^ Even if women are paid by police as we found in a qualitative study of FSW in Baltimore, the power imbalance and threat of arrest are omnipresent.^11^ Some police may also be considered clients who “pay” by not arresting the FSW with whom they have sex.^13,14^ Despite these existing findings, little is presently understood about the frequency with which FSWs take police as clients or its relationship with other structural HIV risk factors and STI infection.

In considering the police-as-client relationship, we started from an understanding of FSW-police social relations as shaped and driven by unequal gendered, economic and power relations, rooted in the multiple, overlapping marginalized identities many FSWs hold.^15^ However, in viewing this relationship, we seek to avoid the dichotomy of FSWs' assumed powerlessness in the face of overwhelming structural forces^16,17^ against the idealized picture that women's (particularly those adversely impacted by poverty and illicit drug use) involvement in sex work is one of purely individual choice.^18^ Examining police as client seeks to specifically consider police power over FSWs' presumed agentic decision making around these clients, even in the context of criminalization, immediate economic need, and safety. Structuration^19^ is a useful theoretical lens, whereby police as client can be viewed as a process of everyday practices between FSWs and their clients (including negotiating price and condom use), but one that may be considerably constrained by a broader context of criminalization and abuse of police power. This broader context has been characterized in a growing body of literature^20^ as a mechanism through which structural violence operates, whereby “unequal power” shapes “unequal life chances.”^21^ However, the police-as-client relationship manifests as “everyday violence”’^22^ wherein the seemingly every day social practice of police as client may in fact represent the normalization and internalization of another form of abusive police power.

Grounded in theory, the present study aims to longitudinally explore the prevalence and correlates of having police as clients among a cohort of street-based FSWs in Baltimore, Maryland.

## METHODS

### Setting

This study was conducted in Baltimore City, Maryland, where buying and selling of sex is criminalized. In 2016, the Department of Justice investigated the Baltimore Police Department and documented police extortion of sex from FSWs within and outside of custody in a report that fueled the establishment of a consent degree.^23^ In 2018, a Maryland Bill was passed stating that a law enforcement officer may not engage in sexual contact, vaginal intercourse, or a sexual act with a person in their custody. The law imposes a maximum 3-year prison sentence and a $3000 US dollars fine on officers who violate the statute.^24^

### Recruitment

Between April 2016 and January 2017, an observational prospective cohort of sex workers was recruited for the SAPPHIRE study, a mixed-methods study in Baltimore, Maryland, that also included a prior qualitative police ethnography phase. The study examined the role of police on the HIV risk environment of street-based FSW over time. Participants were followed up for 12 months with study visits every 3 months, resulting in 5 study visits total. Detailed methods have been published previously.^9,12,25^

Women were approached on the street in specific time-location sampling frames, and interested women were screened in the study van. Eligibility criteria were the following: (1) age ≥15 years; (2) sold or traded oral, vaginal, or anal sex “for money or things like food, drugs or favors”; (3) picked up clients on the street or at public places ≥3 times in the past 3 months; and (4) willing to undergo STI and HIV testing. After informed consent, participants completed a standardized interviewer-administered 50-minute computer-assisted personal interview and STI/HIV testing at baseline and follow-up visits. Participants were permitted to complete any study visit for which they were in the eligibility period, regardless of missed previous visits. Participants received $70 US dollars for baseline and 12-month visits, and $45 for 3-, 6-, and 9-month follow-up visits. Overall 3-, 6-, 9-, and 12-month retention rates were 81%, 74%, 76%, and 73%, respectively, after excluding participants with experiences/outcomes that precluded follow-up participation (e.g., deceased and incarcerated). The study was approved by the Johns Hopkins Bloomberg School of Public Health Institutional Review Board.

### Measures

Sociodemographic, structural vulnerability, and sex work characteristics including age, race/ethnicity, education, homelessness, financial and food insecurity, length of time doing sex work, and frequency of recent sex work were captured on the baseline survey, informed by our previous research.^26,27^ Financial insecurity was defined as having no income from full- or part-time jobs and absence of savings in the past 3 months. Food insecurity was defined as going hungry at least once a week in the past 3 months. Both were collected at baseline only.

#### Policing and Police Encounters

Baseline and follow-up computer-assisted personal interview surveys included items regarding police interactions. The primary outcome, having police as clients in the past 3 months, was assessed based on responses to the question, “How many clients in the last 3 months were police officers?” Respondents were instructed to only include officers they had voluntary oral, vaginal, or anal sex with and paid them for sex. At each time point, women who answered ≥1 were coded as having had any police as clients. To better understand the underlying power dynamics and emotional decision making of FSW reporting voluntary sex with a police officer, those who reported any police as clients were asked follow-up questions about condom use, having sex with police clients because of fear of arrest, and how many police clients they considered “regular” clients.

Respondents were asked about other recent experiences with law enforcement. Unless otherwise noted hereinafter, “recent” was defined as past 12 months at baseline and as past 3 months at all follow-up points, and items were considered as binary (yes/no) variables. We used 2 aggregate measures of experiences with police: one of routine police patrol/enforcement practices and the other of egregious police practices.^9^ The first measure captured which of the following police patrol practices a woman had recently experienced: police asking the woman to move on from a specific location, conducting a routine stop, conducting a search of the woman or her property, confiscating drugs or drug paraphernalia, and confiscating condoms. For patrol practices, the recall period was 3 months at all assessment points. The measure of egregious police practices captured recent experiences of the following abusive practices outside the scope of law enforcement: verbal or emotional harassment, sexual harassment (defined as “making suggestive sexual comments or advances without following through”), physical violence, damage of property, and acceptance of money or other goods in exchange for no arrest. Each respondent reported whether or not she had recently experienced each of these items, and the number of “yes” responses to patrol practice questions and egregious practice questions was totaled into 2 aggregate measures for each time point: total number of different police patrol practices recently experienced and total number of different egregious police practices recently experienced. Given our focus on sexual encounters between the police and FSW, we considered being pressured to have sex in exchange for no arrest or being forced to have unwanted vaginal or anal sex through the use of physical force (rape) as its own separate variable and did not include it in the aggregate abusive measure. This measure pertained to all police, not just those referred to as “clients.” Respondents also reported if they had recently changed occupational-related behaviors as a result of a fear of police, including avoiding carrying condoms and rushing client negotiations.

#### Substance Use

Lifetime (baseline only) and recent (past 3 months) drug use was ascertained, exploring a range of types (e.g., heroin, crack cocaine, and prescription opioids) and routes of drug administration (e.g., snorting, smoking, and injection), informed by measures used in earlier studies of the study population.^26,27^ Binary variables were generated for daily use of powdered/crack cocaine, heroin, and drug injection at each visit.

#### Violence

Recent (past 3 months) client violence was defined as pressured or forced vaginal or anal sex, physical violence, or being threatened or hurt with a weapon in the past 3 months by paying sex work clients, excluding police officers.^7^

#### Health Outcomes

Sexually transmitted infection tests were conducted at each study visit via participant-administered vaginal swabs that were sent to the Baltimore City Health Department for chlamydia and gonorrhea (STI) testing.^28^ HIV status over time was ascertained via study staff-administered rapid oral OraQuick.

### Statistical Analysis

The SAPPHIRE cohort consisted of 250 cisgender FSWs. Women who did not answer the police client item (n = 4 at baseline only) were excluded from cross-sectional baseline analyses, resulting in n = 246 women in baseline analyses and n = 250 FSWs in the longitudinal sample. We conducted descriptive analyses comparing the baseline characteristics of women who did and did not report police clients using Pearson χ^2^ tests (Fisher exact tests used for variables with cells containing <5 observations, as noted in Table 1) for binary and categorical variables, and *t* tests for continuous variables (Table 1). The cohort completed 917 study visits; however, only visits where recent (past 3 months) sex exchange was reported were considered for analysis, resulting in data from n = 752 visits. Use of complete case analysis resulted in data from 711 visits included in the final multivariate model.

**TABLE 1:** Baseline Characteristics of the SAPPHIRE Cohort, Stratified by Having Police Clients in the Past 3 Months (n = 246)*

		Police Clients, Past 3 mo		*P*
	Total (n = 246)	None (n = 205; 83.3%)	1+ (n = 41; 16.7%)	
Structural vulnerability/demographics				
Age, mean (SD), y	35.77 (9.00)	35.98 (9.20)	34.71 (7.90)	0.409
Race/ethnicity				0.090
White	162 (65.9%)	135 (65.9%)	27 (65.9%)	
Black	57 (23.2%)	51 (24.9%)	6 (14.6%)	
Hispanic or other	27 (11.0%)	19 (9.3%)	8 (19.5%)	
Highest education level				0.689
Less than grade 12	131 (53.3%)	108 (52.7%)	23 (56.1%)	
High school/GED	115 (46.7%)	97 (47.3%)	18 (43.9%)	
Homelessness, past 3 mo	167 (67.9%)	138 (67.3%)	29 (70.7%)	0.597
Financial instability, past 3 mo	203 (82.5%)	171 (83.4%)	32 (78.1%)	0.409
Food insecurity more than once a week, past 3 mo	133 (54.1%)	108 (52.7%)	25 (61.0%)	0.331
Substance use				
Daily cocaine use	156 (63.4%)	130 (63.4%)	26 (63.4%)	1.00
Daily heroin use	173 (70.3%)	139 (67.8%)	34 (82.9%)	0.053
Daily drug injection	139 (56.5%)	113 (55.1%)	26 (63.4%)	0.328
Sex work characteristics and clients				
≤5 y in street-based sex work at baseline	118 (48.0%)	100 (48.8%)	18 (43.9%)	0.568
Daily sex work, past 3 mo	164 (66.7%)	133 (64.9%)	31 (75.6%)	0.183
30+ clients in the past 3 mo	136 (55.3%)	110 (53.7%)	26 (63.4%)	0.251
Minor at sex work entry	52 (21.1%)	43 (21.0%)	9 (22.0%)	0.889
Physical or sexual violence by paying clients	84 (34.2%)	65 (31.7%)	19 (46.3%)	0.071
Recent policing/police encounters				
Recent arrest	113 (45.9%)	85 (41.5%)	28 (68.3%)	0.002
Pressured or forced sex	7 (2.9%)	3 (1.5%)	4 (9.8%)	0.016^†^
Routine patrol practices				
No. patrol practices experienced, mean (SD)	2.00 (1.46)	1.87 (1.50)	2.63 (1.07)	0.002
• Asked to move along	164 (66.9%)	130 (63.7%)	34 (82.9%)	0.017
• Routine stop	159 (64.6%)	124 (60.5%)	35 (85.4%)	0.002
• Searched person or property	97 (39.4%)	74 (36.1%)	23 (56.1%)	0.017
• Confiscated drugs/drug paraphernalia	63 (25.6%)	47 (22.9%)	16 (39.0%)	0.031
• Confiscated condoms	9 (3.7%)	9 (4.4%)	0 (0.0%)	0.363^†^
Abusive policing practices				
No. egregious practices experienced, mean (SD)	0.56 (0.84)	0.46 (0.74)	1.02 (1.13)	<0.001
• Verbal/emotional harassment	74 (30.1%)	57 (27.8%)	17 (41.5%)	0.082
• Sexual harassment	44 (17.9%)	25 (12.2%)	19 (46.3%)	<0.001
• Damaged property	12 (4.9%)	9 (4.4%)	3 (7.3%)	0.427
• Physical violence	4 (1.6%)	2 (1.0%)	2 (4.9%)	0.131^†^
• Accepted money/goods in exchange for no arrest	3 (1.2%)	2 (1.0%)	1 (2.4%)	0.423^†^
Because of fear of police				
• Avoided carrying condoms (n = 245)	34 (13.9%)	25 (12.3%)	9 (22.0%)	0.101
• Avoided carrying ID (n = 243)	74 (30.5%)	61 (30.2%)	13 (31.7%)	0.848
• Moved to unfamiliar location (n = 245)	55 (22.4%)	48 (23.5%)	7 (17.1%)	0.366
• Rushed negotiations with clients	135 (54.9%)	108 (52.7%)	27 (65.9%)	0.122
Health outcomes				
Positive test for chlamydia/gonorrhea infection (n = 235)	44 (18.7%)	30 (15.2%)	14 (36.8%)	0.002
Positive test for HIV infection (n = 245)	13 (5.3%)	10 (4.9%)	3 (7.3%)	0.529

*Excluding those who did not answer question about having police as clients (n = 4).

^†^*P* value associated with Fisher exact test.

Using general estimating equations to account for intraperson correlations over time, we explored longitudinal bivariate associations between having police as clients and independent variables described earlier. No time indicators were included in the model, such that results represented cross-sectional correlates, rather than change, over time. We conducted multivariate logistic analysis, using general estimating equation, to test a full model and several alternative models using subsets of all covariates that were significant at the *P* < 0.20 level in bivariate models, along with age and race/ethnicity. Because it is unlikely that any individual type of police interaction occurs in isolation of other police interactions and cumulative exposure to a range of different practices may impact the FSWs' risk environment, we only used aggregate egregious police practice and police patrol practice scales (rather than the individual police variables) in the multivariate analysis. Final covariate selection was based on model fit, as measured by quasi-information criterion value, and prior research. All analyses were conducted using Stata/MP 15.1 (College Station, TX).

## RESULTS

Baseline prevalence of structural vulnerability, demographic, substance use, sex work, police encounters, and health variables for the SAPPHIRE cisgender FSW cohort, stratified by whether or not women reported recent police as clients, are displayed in Table 1. At baseline, mean age in the overall sample was 35.8 years, 65.9% were White, and 53.3% had less than a 12th grade education. Daily heroin, cocaine, and drug injection use was reported by 70.3%, 63.4%, and 56.5% of respondents, respectively. Nearly half of FSW reported past 3 month arrest. Overall, 24.8% (n = 62) reported recent police clients at any time point. Table 2 presents prevalence of reporting recent police clients and related variables at baseline and all follow-up points. At baseline, 16.7% (n = 41) of respondents reported recent police clients, with those who reported police clients having an average of 2 different police clients, comprising an average of 11% of total clients. Most FSWs (76%) categorized their police clients as “regular” clients. At follow-up points, the proportion of respondents who reported recent police clients ranged from 7.9% to 14.3%, and mean proportion of their clients who were police ranged from 8% to 13%.

**TABLE 2:** Prevalence and Characteristics of Police Clients in SAPPHIRE Cohort Over Time (n = 250)

	Baseline	3 mo	6 mo	9 mo	12 mo	Total Over Time (Reported at Any Time Point)
Total in cohort who sold sex in past 3 mo	246*	155	131	119	101	—
Any police clients, past 3 mo	41 (16.7%)	21 (13.6%)	13 (9.9%)	17 (14.3%)	8 (7.9%)	62 (24.8%)
Among those with any police clients						
• No. police clients, mean (SD)	1.98 (1.39)	2.33 (1.28)	2.23 (1.36)	1.71 (0.99)	1.38 (0.52)	—
• Proportion of clients that were police, mean (SD)	0.09 (0.11)	0.13 (0.15)	0.11 (0.10)	0.08 (0.12)	0.11 (0.14)	—
• Any police clients were regular clients	31 (75.6%)	18 (85.7%)	11 (84.6%)	9 (52.9%)	6 (75.0%)	48 (77.4%)
• Always used condoms with police clients	34 (82.9%)	17 (81.0%)	11 (84.6%)	12 (70.6%)	5 (62.5%)	54 (87.1%)
• Police clients ever refused condoms	4 (9.8%)	4 (19.1%)	2 (15.4%)	2 (11.8%)	1 (12.5%)	10 (16.1%)
• Had sex with police client out of fear of arrest	7 (24.1%)^†^	9 (42.9%)	6 (46.2%)	5 (29.4%)	Not asked	21 (33.9%)

*Excluding those who did not answer question about having police as clients (n = 4).

^†^Denominator is 29 because the question was added late.

At baseline, FSWs with recent police as clients were significantly more likely to report recent arrest (68.3% vs. 41.5%; *P* = 0.002). The mean (SD) number of recent police patrol practices experienced was significantly higher among those with police as clients (2.63 [1.07] vs. 1.87 [1.50]; *P* = 0.002), and they were significantly more likely to report experience of all patrol practices other than condom confiscation. The mean (SD) number of recent egregious police practices experienced was also significantly elevated among the group of women with recent police as clients (1.02 [1.13] vs. 0.46 [0.74]; *P* < 0.001). Among the egregious practices, women with police as clients were significantly more likely to report recent sexual harassment by police (46.3% vs. 12.2%; *P* < 0.001). They were also more likely to have been pressured or forced to have sex with police (9.8% vs. 1.5%; *P* = 0.016) and to test positive for STI (36.8% vs. 15.2%; *P* = 0.002). Reports of recent client-perpetrated violence (46.3% vs. 31.7%, *P* = 0.071) and daily heroin use (82.9% vs. 67.8%; 0.053) were more prevalent among FSWs with police as clients, but the difference fell short of significance. At baseline, they did not differ significantly from other FSWs on any other demographic, structural vulnerability, substance use, or fear-motivated behavior change measures (all, *P* > 0.05).

Unadjusted and adjusted odds ratios (aORs) are presented in Table 3. In the final adjusted multivariate model, factors that remained independently associated with recent police clients were recent arrest (aOR, 1.76; 95% confidence interval [CI], 1.03–2.99; *P* = 0.037), coerced or forced sex by police (aOR, 4.47; 95% CI, 1.79–11.12; *P* = 0.001), higher number of egregious police practices experienced (aOR, 1.77; 95% CI, 1.38–2.29; *P* < 0.001), and having an STI infection (aOR, 2.43; 95% CI, 1.46–4.04; *P* = 0.001).

**TABLE 3:** Bivariate and Multivariate Longitudinal Correlates of Having Police Sex Work Clients in the SAPPHIRE Cohort (n = 250)

	Unadjusted Odds Ratio (95% CI)	*P*	Adjusted Odds Ratio* (95% CI)	*P*
Structural vulnerability/demographics				
Age, y^†^				
18–29	Reference		Reference	
30–39	1.70 (0.90, 3.21)	0.105	1.87 (0.97–3.60)	0.063
40+	0.90 (0.42–1.93)	0.780	1.43 (0.64–3.21)	0.385
Race/ethnicity^†^				
White	Reference		Reference	
Black	0.60 (0.29–1.22)	0.158	1.19 (0.54–2.65)	0.668
Hispanic or other	1.25 (0.58–2.70)	0.562	1.17 (0.50–2.75)	0.725
Highest education level^†^				
Less than grade 12	Reference		—	—
Graduated high school/GED	1.15 (0.79–1.66)	0.473	—	—
Homeless, past 3 mo^§^	1.51 (1.02–2.24)	0.041	0.85 (0.50–1.45)	0.551
Financial Instability at baseline^†§^	—	—	—	—
Food insecurity more than once a week at baseline^†§^	—	—	—	—
Substance use				
Daily cocaine use^‡^	1.49 (0.87–2.54)	0.143	—	—
Daily heroin use^‡^	1.65 (0.95–2.85)	0.073	—	—
Daily drug injection^§^	2.12 (1.23–3.66)	0.007	1.38 (0.80–2.38)	0.247
Sex work characteristics and clients				
≤5 y in street-based sex work at baseline	0.99 (0.57–1.71)	0.965	—	—
Daily sex work, past 3 mo^†^	—	—	—	—
30+ clients in the past 3 mo	1.65 (1.12–2.43)	0.012	1.41 (0.87–2.30)	0.167
Minor at sex work entry	0.95 (0.46–1.94)	0.882	—	—
Physical or sexual violence by paying clients^§^	1.69 (1.16–2.46)	0.006	0.88 (0.53–1.45)	0.604
Recent policing/police encounters				
Recent arrest^‡^	2.24 (1.41–3.55)	0.001	1.76 (1.03–2.99)	0.037
Pressured or forced sex^‡^	6.32 (2.84–14.10)	<0.001	4.47 (1.79–11.12)	0.001
Routine patrol practices				
No. patrol practices experienced, mean (SD)^§^	1.61 (1.38–1.88)	<0.001	1.13 (0.95–1.33)	0.157
• Asked to move along^§^	4.02 (2.30–7.03)	<0.001	—	—
• Routine stop^§^	3.68 (2.19–6.19)	<0.001	—	—
• Searched person or property^§^	3.25 (2.12–4.97)	<0.001	—	—
• Confiscated drugs/drug paraphernalia^§^	2.13 (1.21–3.74)	0.009	—	—
• Confiscated condoms^§^	1.22 (0.60–2.47)	0.579	—	—
Abusive policing practices				
No. egregious practices experienced, mean (SD)^‡^	2.05 (1.67–2.53)	<0.001	1.77 (1.38–2.29)	<0.001
• Verbal/emotional harassment^‡^	2.88 (1.84–4.51)	<0.001	—	—
• Sexual harassment^‡^	9.26 (5.60–15.31)	<0.001	—	—
• Damaged property^‡^	2.33 (1.26–4.29)	0.007	—	—
• Physical violence^‡^	5.39 (2.12–13.69)	<0.001	—	—
• Accepted money/goods in exchange for no arrest^‡^	2.40 (0.96–6.02)	0.062	—	—
Because of fear of police				
• Avoided carrying condoms^‡^	2.38 (1.52–3.72)	<0.001	—	—
• Avoided carrying ID^‡^	1.51 (0.97–2.33)	0.068	—	—
• Moved to unfamiliar location^‡^	1.59 (1.03–2.45)	0.036	—	—
• Rushed negotiations with clients^‡^	2.28 (1.38–3.77)	0.001	—	—
Health outcomes				
Positive test for chlamydia/gonorrhea infection	2.26 (1.42–3.60)	0.001	2.43 (1.46–4.04)	0.001
Positive test for HIV infection^¶^	—	—	—	—

*Adjusted for all variables listed.

^†^Item collected at baseline only.

^‡^Recall period 12 months at baseline and 3 months at other visits.

^§^Recall period 3 months at all visits.

^¶^Not measured at all visit.

## DISCUSSION

The study is one of the first to document the role of egregious police practices and prevalent STIs in association with having police as clients, among a longitudinal cohort of street-based FSW, and provides novel insights into an important aspect of FSWs' social relations with police in the context of sex work criminalization. We found that a relatively high percentage of FSWs reported having clients who were police officers during a 12-month period (25%), of whom most (77.4%) were “regulars,” meaning repeat clients with whom they had an ongoing arrangement. The fact that participants were asked separately about consensual police client relations and that many went on to become regular clients could be indicative of FSWs' potentially underexplored agency/resistance and retention of power and control over some sexual relations with police. To better explore the volitional nature, we further ascertained whether FSWs who reported having police as clients had sex with police as clients because of fear of arrest. Results show that of those FSWs who ever reported police as clients, 34% reported having had sex with the officer because of fear of arrest. This psychological fear of arrest by FSW is distinct and likely to be more ubiquitous from the actual threat of arrest being invoked by an officer to have sex (explicit sexual coercion). However, of those FSWs who said they had sex with police as clients out of fear of arrest at any time point, 41% also separately reported being forced or pressured by police to have sex, underscoring the multiple layers of constraints on FSWs' ability to negotiate sex with police. Our study thus provides evidence of a gradation by which the police-FSW power imbalance manifests. Specifically, we have evidence of police as clients without fear of arrest, suggesting higher degree of volition, evidence of fear of arrest within the police-as-client dynamic, which illustrates exploitation of power and instances of force and pressure for sex by police, which clearly illustrates sexual violence within this dynamic. This finding strongly suggests that FSWs' broader experience of structural violence, operationalized through the intersection of sex work criminalization and egregious police practices, inevitably shapes the police-as-client interaction. Police as client, far from being a more equal association between officers and FSWs, instead seems to embody a form of “everyday violence”: that both normalizes and legitimizes police power and structural violence. The results support existing global research of the negative outcomes of criminalization,^5^ underscoring the need for decriminalization of sex work.

Findings also showed that FSWs who had a history of more abusive interactions with the police were more likely to have had police as clients. For each additional egregious police encounter reported, the odds of having police as clients nearly doubled. At baseline, women who reported recent police as clients were nearly 4 times more likely to have experienced recent sexual harassment by police and roughly 5 times more likely to report pressure or forced sex with police. Female sex workers who had police as clients were also more likely to engage in occupational HIV and STI risk behaviors, including rushing client negotiations and not carrying condoms for fear of interacting with police. These results point to the police-as-client association embodying and being a proxy for, rather than mitigating, FSWs' experience of structural violence. Instead of FSWs being able to use this association to offset additional risk (e.g., police harassment) or provide additional protection (e.g., from forms of workplace-related violence), we see that this association is a marker for increased police interactions. This in turn presents the opportunity for police abuse including harassment, property damage, and physical and sexual violence, as well as engagement in HIV/STI risk-related behaviors. These results support existing evidence in this setting^9,11,23^ and international settings^1,6,29^ that point to the frequently involuntary nature of FSWs' relations with police, wherein police are able to capitalize on elements of women's risk environment (e.g., criminalization of sex work and illicit drug use, and immediate need for illicit drugs) to victimize women and commit sexual abuse. In addition, we found that in bivariate analysis, daily injection drug use was strongly associated with having police as clients, suggesting the increased risk conferred by drug use, yet the association became attenuated in the multivariable analysis, suggesting the distal nature of drug use compared with regular police interactions (e.g., arrest) for taking police as clients.

Finally, we found that those FSWs with a positive result for STI were more likely to have had a police as client. This result supports the conclusion that FSWs' STI risk is embedded in a complex web of structural violence that extends to the police as client and the power imbalances that enable and sustain these interactions. Sexually transmitted infections persisted over the study period even with STI screening and referrals at each of the 5 study time points. Although the city STD clinic began conducting outreach to FSWs on the heels of a syphilis outbreak in 2017, tailored, comprehensive, STI interventions are needed along with police policy changes and systems of accountability to reduce the egregious police behaviors that potentiate women's already elevated STI risk.

These findings are subject to a number of limitations. First, it is difficult with a purely quantitative study to do justice to the influence of structural factors that shape women's decision making around taking a police officer as a client. Second, although we used data from multiple time points in a longitudinal cohort study, our findings represent cross-sectional correlates over time and do not assess temporality or causality. Although we found a highly significant association between STI and having police as clients, STI was not the “outcome” in our multivariate model, so all we can conclude is that prevalent STI infection is associated with police as clients, not that the latter is a cause of the former. All measures, with the exception of HIV and STI tests, were self-reported. Furthermore, we did not have objective documentation if women received STI treatment after a previous diagnosis, thereby reducing our ability to know if subsequent positive STI results were prevalent or incident. Given the sensitive nature of questions around sex, violence, and policing, social desirability or response bias is possible. In addition, our targeted sampling may not have yielded a truly representative sample of FSWs in Baltimore. Less than one-quarter of our sample identified as Black, which is surprising given that more than 60% of Baltimore's overall population is Black.^30^ This might be due to more Black FSWs selling sex in off-street locations, perhaps to avoid overpolicing and law enforcement discrimination of Black communities in Baltimore.^23,26^

Prevalent STI was a significant driver of police as clients among a sample of street-based FSWs. Understanding police as clients necessitates recognition of deeply unbalanced power dynamics that shape FSWs' decision making, enabled by a structural context of criminalization that rests on gender inequity and violence. Structuration theory hypothesizes that, although structure influences individual agency, in this case police power, individuals also have the ability to use their agency to maintain or adapt to those structures,^19^ even in the presence of great power imbalances. Whether police as client represents more agentic social relations with police that are volitional in nature and challenge the usually unequal police-FSW power dynamic has, until now, been largely underexplored. Study results are hugely significant in that they provide evidence that the police-as-client relation is common, an important marker for broader police abuse of power aided by a situational context of criminalization and associated with prevalent STIs. Beyond pointing once again to the urgent need for decriminalization, such evidence should prompt police departments to better investigate, identify, and punish police officers who engage in sex on- or off-duty with FSWs. At an organizational level, these findings go further, prompting recognition within policing establishments that, rather than being a harmless norm of police culture, this practice is an important indicator of broader police abuse and violations of women's political, economic, social, and cultural rights that requires urgent attention.
